# Assessing disparity in mortality rates for ischemic heart disease using CDC-WONDER database: A retrospective analysis

**DOI:** 10.21542/gcsp.2025.14

**Published:** 2025-02-28

**Authors:** Nithin Karnan, Abdirahman Hassan Abdirahman, Gayathri Kilaru, Asis Reet Kaur, Kaustav Majumder, Layla Al Safadi Abou Al Fadel

**Affiliations:** 1 K.A.P. Viswanatham Medical College, Tiruchirapallii, Tamil Nadu, India; 2 Southern Medical University, Guangzhou, Guangdong, China; 3 Priyanka Nursing Home, Vijayawada, Andhra Pradesh, India; 4 International Medical University, Kuala Lumpur, Malaysia; 5 R.G. Kar Medical College and Hospital, Kolkata, West Bengal, India; 6 Khalifa University, Abu Dhabi, United Arab Emirates

## Abstract

Introduction: Ischemic heart disease remains a leading global cause of death, impacting healthcare systems worldwide. In 2019, 32% of global deaths were estimated to be due to cardiovascular events, accounting for approximately 17.9 million people worldwide. Despite technological advances improving diagnosis and treatment, disparities persist, especially between urban and rural areas.

Methods: We conducted a retrospective study using the CDC WONDER database extracted on 17th March 2024, ICD-10 code: I20–I25 (Ischemic Heart Disease). We selected the years 1999–2020: Underlying Cause of Death by Bridged-Race Categories divided based on rural and urban deaths, based on 2013 urbanization classification, and grouped by age (10-year age range), gender, and race.

Results: Our research indicates a consistent decline in ischemic heart disease mortality across urban and rural regions within all demographic groups from 1999 to 2020. However, a notable exception occurred in 2019 when mortality rates increased in both urban and rural settings. Notably, throughout the entire period under study, rural areas consistently exhibited higher mortality rates compared to their urban counterparts.

Conclusion: This study sheds light on the big disparity in mortality rates due to ischemic heart disease in urban versus rural areas in all groups of age, gender, and race. We hope our findings prompt further research to determine the causes of this discrepancy and public health interventions to address it.

## Introduction

Ischemic heart disease remains a leading cause of mortality worldwide, exerting a substantial burden on worldwide public healthcare systems, with approximately 17.9 million people succumb to cardiovascular events globally, and representing 32% of global deaths, as reported by the World Health Organization (WHO, 2019)^1,2^.

Despite recent advancements in technologies resulting in improvement in diagnostic and treatment process, there are still many disparities that continue to persist which continue to affect the prevalence and outcomes of ischemic heart disease, especially across different geographic areas, such as rural and urban areas^3^. There are many factors that may contribute to such disparities, for example, lifestyle differences, education and knowledge about the disease, access to healthcare, socioeconomic factors, among others^4,5^. Moreover, certain geographical areas may have limited resources, for example a retrospective cross sectional study revealed that those from rural areas are less likely to undergo cardiac catheterization within 30 days of initial presentation^6^. Thus, if equipped with this knowledge, healthcare providers would be able to customize strategies to address the respective needs of the geographical area. This in turn, would also help to promote healthcare equity, and improve access and delivery of healthcare services to underserved rural areas. Collectively, this focused intervention and planning process could optimize the healthcare system, and hence reduce the burden of ischemic heart disease mortality.

Rural areas often face significant barriers in accessing healthcare compared to urban regions, which directly impacts disease reporting and medical research. Limited healthcare infrastructure, fewer medical facilities, and a shortage of trained professionals in rural settings make it challenging for residents to receive timely diagnosis and treatment. Geographic isolation, inadequate transportation, and financial constraints further exacerbate these issues^7,8^. Consequently, diseases in these areas are often underreported, leading to an incomplete understanding of their prevalence and progression. The lack of robust patient data from rural populations hampers the ability to conduct comprehensive studies, develop targeted interventions, and allocate resources effectively, thereby perpetuating health disparities between rural and urban communities^8^.

There is a lack of such research on disparities related to ischemic heart disease mortality in urban-rural communities, and thus, this study aims to fill in the knowledge gap and discover the underlying factors contributing to such disparities.

## Aims and objectives

This study aims to assess the disparity in mortality rates for the ischemic heart disease between urban and rural areas over the past twenty years using data from the CDC-WONDER database. In this retrospective analysis this study plans to look at various parameters like age, gender, and race to understand the variations of mortality rate among these parameters.

Aside from merely looking at the numbers, this study also wants to understand why these differences exist between urban and rural areas. It will explore various factors that might play a role in the disparity in mortality rates.

## Methods

A retrospective original research study was conducted utilizing the Centers for Disease Control and Prevention Wide-ranging Online Data for Epidemiologic Research database (CDC WONDER). Data extraction was performed on March 17, 2024. As the research involved non–human participants and utilized deidentified public data from CDC-WONDER, no ethics committee approval was deemed necessary^9^.

The data collected pertained to underlying causes of death, specifically focusing on the years 1999 to 2020 and categorized under the Bridged-Race Categories. The International Classification of Diseases, Tenth Revision (ICD-10) code I20–I25, which corresponds to Ischemic Heart Disease, was selected for analysis. For classification purposes, the Metropolitan 2013 classification system was utilized, distinguishing between Urban/Metropolitan and Rural/Non-Metropolitan areas^10^. The data was further grouped by age grouping (10-year age ranges), gender (female, male), and race (White, African-American/Black, American Indian or Alaska Native, Asian or Pacific Islander).

The data thus collected was exported to Microsoft Excel for further analysis. Statistical analysis was conducted using R, with the graphical representations generated using GGPlot 2: Elegant Graphics for Data Analysis, published by Springer-Verlag New York in 2016. Binomial tests were applied to identify associations between demographics and mortality rates.

**Table 1:** Absolute number of reported mortalities in Urban and Rural areas due to IHD from 1999–2020 as per 2013 Urbanization Classification.

Type	n (%)
***Urban (Metropolitan area)***	***7,345,561***
Large Central Metropolitan	2,617,404 (35.6%)
Large Fringe Metropolitan	2,035,004 (27.7%)
Medium Metropolitan	1,821,673 (24.8%)
Small Metropolitan	871,480 (11.9%)
***Rural (non-metropolitan area)***	***1,763,083***
Micropolitan	970,603 (13.2%)
Non-core	792,480 (44.9%)

## Results

Aggregate data of mortality due to ischemic heart disease from 1999–2020 was obtained from CDC WONDER database.

Table 1 shows that the total number of deaths from ischemic heart disease in urban areas to be 7,345,561; of which 35.6% of deaths occurred in Large Central Metropolitan (*n* = 2,617,400), 27.7% deaths in Large Fringe Metropolitan (*n* = 2,035,004), 24.8% in Medium Metropolitan (*n* = 1,821,673), and 11.9% in Small Metropolitan (*n* = 871,480).

In contrast, the total number of deaths due to ischemic heart disease in rural areas was 1,763,083. Of these, 13.2% (*n* = 970,603) were reported in the Micropolitan area, whereas 44.9% (*n* = 792,480) were Non-core (non-metropolitan).

**Figure 1.**
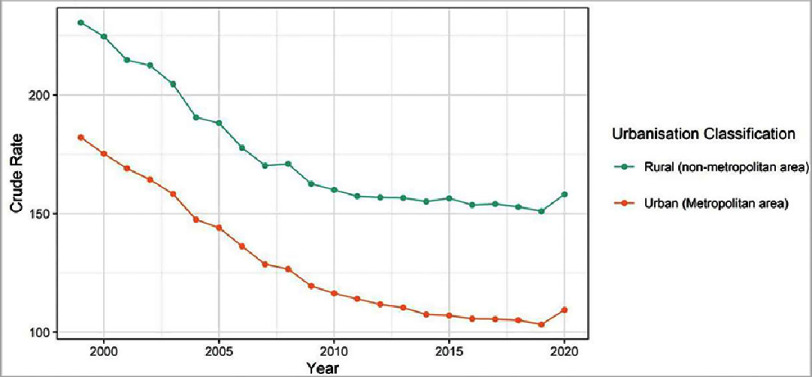
Trends in urban versus rural mortality due to ischemic heart disease calculated in crude rate per 100,000 population.

Figure 1 shows that the rural mortality (solid green line) is consistently higher than urban mortality (solid orange line) in all years from 1999–2020. However, the mortality rate has been decreasing in the years between 1999 and 2020 for both urban and rural areas except from 2019 to 2020 when it has slightly increased in both urban and rural areas. There was also a minor spike in mortality in just rural, not urban, areas in the year 2015.

Table 2 depicts the mortality rates due to Ischemic Heart Disease in Urban and rural areas based on age, gender and race. According to age groups, mortality rates in urban areas were found to be highest amongst 75–84 year olds at 8397.26 deaths per 100,000; while among rural areas, 85 and above had the highest mortality rates (2798 per 100,000). Males had higher mortality rates in both urban (141 per 100,000) and rural (195 per 100,000) areas. According to race, White individuals had the highest mortality rates in both urban (140 per 100,000) and rural (184 per 100,000) areas. Table 2 depicts that for the age group 85 and above rural areas have higher mortality (2798 per 100,000) than its urban counterparts (2713 per 100,000).

**Table 2:** Mortality rates due to ischemic heart disease in urban and rural areas based on age, gender and race.

Variables	Urban		**Mortality rate (per 100,000)**	Rural		**Mortality rate (per 100,000)**	Binomial test
	Mortality	Total population		Mortality	Total population		*P* value
***Age Groups***							
<1 year	324	74818291	**4**	45	12095361	**0.37**	0.334
1–4 years	120	299561638	**0.4**	28	49008275	**0.057**	0.069
5–14 years	264	770126283	**0.34**	65	131095742	**0.05**	0.004^*^
15–24 years	2258	799755615	**2.82**	588	136038105	**0.43**	<0.001^*^
25–34 years	17528	803054375	**21.83**	4558	117033859	**3.89**	<0.001^*^
35–44 years	102776	803916304	**127.84**	27718	127369780	**21.76**	<0.001^*^
45–54 years	394402	788609639	**500.12**	100188	138965498	**72.1**	<0.001^*^
55–64 years	838481	639136856	**1311.9**	214798	127286485	**168.75**	<0.001^*^
65–74 years	1270159	417988177	**3038.74**	330411	92469123	**357.32**	<0.001^*^
75–84 years	2050475	244183949	**8397.26**	491587	54320144	**904.98**	<0.001^*^
85+ years	2668224	98324522	**2713.691**	593046	21189280	**2798.80**	<0.001^*^
***Gender***							
Male	3959173	2815055490	**140.6**	980438	502292400	**195.19**	<0.001^*^
Female	3386388	2924420159	**115.8**	782645	504579252	**155.11**	<0.001^*^
***Race***							
American Indian or Alaska Native	22671	62750775	**36.13**	19971	25610416	**77.98**	<0.001^*^
Asian or Pacific Islander	175580	359973366	**48.78**	6829	11940089	**57.19**	<0.001^*^
Black or African American	853441	830766851	**102.73**	112691	88267994	**127.67**	<0.001^*^
White	6293869	4485984657	**140.30**	1623592	881053153	**184.28**	<0.001^*^

**Notes.**

* *P*-value less than 0.005.

Table 2 shows that higher mortality due to Ischemic Heart Disease was significantly associated with ages 5 years and older, regardless of gender and race (*P*-value less than 0.05) i.e, there was a significant association in increased mortality for ages 5 years and older in both genders and all races. On the other hand, the data showed no significant difference for the other age groups with regards to mortality from ischemic heart disease (*P*-value 0.334) and (*P*-value 0.069) for <1 year olds and 1–4 year olds, respectively.

## Discussion

Here, the variation in ischemic heart disease mortality rates over a 22-year period was investigated by a retrospective original research study, using information from the CDC-WONDER database. We found that metropolitan areas generally exhibited higher overall death rates compared to rural areas. Notably, urban locations consistently showed higher numbers of total deaths over the study period. However, within rural regions, mortality rates were significantly elevated in the rural areas, compared to urban areas, among individuals of white racial background, male gender, and those aged 75 and older.

A study conducted by Jones et al., in 2005 via the Behavior Risk Factor Surveillance Survey in the United States, explored the significance of residence location in relation to health infrastructure access and contextual factors impacting health, and highlighted racial disparities in IHD diagnosis, emphasizing the role of residence in influencing health outcomes^11^. The present study found mortality rates to be higher among white individuals, and in rural areas. These findings can be contrasted to the analysis of the National Center for Health Statistics data by Kulshreshtha et al., revealed intriguing trends in IHD death rates between 1999 and 2009, where both black and white individuals experienced similar mortality trends, but the magnitude was higher in urban than rural areas^4^. A shift in the trends was observed in 2007, with rural areas surpassing urban areas in mortality rates, and the disparity persisted, particularly among black individuals residing in rural regions of the Southern USA^4^.

Furthermore, a retrospective analysis of Medicare fee-for-service data by Loccoh et al., highlighted disparities in thrombolysis and endovascular treatment rates for IHD in rural hospitals. Patients presenting with IHD at rural hospitals faced significantly higher mortality rates at both 30 and 90 days post-admission, particularly in critical access hospitals serving remote areas^7^. This could explain the findings of the present study, which observed increased mortality rates in rural areas among various demographic groups, and can also be explained by the study by Minhas et al., who observed that during their study period, hospitalizations for IHD in urban areas experienced a continuous drop between 2004 and 2010, while in rural areas it showed a steady increase, with the in-hospital mortality rate often lower in urban hospitals than in rural hospitals^12^.

The present study compared the mortality rates across gender, 10-year age groups and race. Regarding gender, males were found to have higher mortality rates in both urban and rural areas. This observation can be explained by the study by Shah et al., which found that women are less likely than men to have IHD at any age^13^. However, the authors also noted that this difference narrows with increasing age, and women with IHD have worse clinical outcomes than men in terms of myocardial infarction mortality, all-cause mortality, and reinfarction rates; consequently, there is a possibility that women may be subject to underdiagnosis of IHD, potentially contributing to the observed lower mortality rates^13,14^.

The present study found ages 85 and above to have the highest mortality rates in rural areas. This finding was also noted by Shah et al., and can be explained by the older age at diagnosis and increased rate of comorbid conditions that come with advanced age as the main contributing factors^13^. Contrastingly, within urban areas, the mortality rates were higher in the 75–84 and 65–74 years age groups compared to the 85 and above age groups. The higher mortality rates among the younger age groups in urban areas could be attributed to the higher prevalence of risk factors, such as diet, comorbidities, demographic changes, socioeconomic, and psychosocial factors^15,16^. The findings thus warrant the development of more focused medical interventions to control and prevent IHD and associated mortality.

Regarding race, the present study found White individuals in rural areas to have the highest mortality rates, followed by White individuals in urban areas. This is contrasted by the findings of Holmes et al., who observed that compared to white people, black people between the ages of 45 and 64 have a 5.6-fold higher risk of IHD hospitalization, one-third lower chance of having a cardiac procedure, and an almost two-fold higher risk of IHD death^19^. The difference in findings could be explained by the noted decline in primary care physicians and specialists in rural areas, potentially exacerbating challenges related to accessing follow-up care post-discharge. This diminished availability, coupled with poorer access to crucial services among Black individuals like cardiac rehabilitation and rehabilitative care following strokes, and may contribute to inferior outcomes in rural settings.

The study indicates that both in rural and urban areas, white individuals experience higher mortality rates from ischemic heart disease compared to black individuals, a disparity likely influenced by lifestyle factors, ethnic background, and genetic predisposition. Lifestyle choices such as higher rates of smoking, unhealthy diets, and sedentary behavior, which are more prevalent in some white populations, may elevate the risk of ischemic heart disease. Ethnic background plays a role in shaping health behaviors, cultural attitudes toward healthcare, and access to preventive measures^17^. Additionally, genetic predisposition might influence vulnerability to ischemic heart disease differently across ethnic groups, with certain genetic markers potentially offering protective effects in black populations^18^. However, the disparity also points to complex socio-economic and healthcare dynamics, including variations in healthcare accessibility, disease management, and preventive care utilization, which need further investigation to fully understand and address the observed trends^18,19^.

When compared to White and African American populations, Asian and American Indian populations exhibit significantly lower mortality rates from ischemic heart disease, a difference that may be influenced by lifestyle, genetic factors, and cultural practices. Asian populations, for instance, often adhere to diets rich in vegetables, fruits, and fish, and they tend to have lower rates of obesity and smoking, which are protective against ischemic heart disease. American Indians, though historically facing many health disparities, may benefit from unique community-based health initiatives or genetic factors that mitigate risks for certain heart conditions. Additionally, these groups might have distinct genetic predispositions that confer some level of protection against ischemic heart disease^20^. However, it’s important to note that socio-economic factors and access to healthcare also play a role in shaping these mortality rates, highlighting the complexity of interactions between biology, lifestyle, and environment.

Addressing systemic deficiencies is crucial for ensuring equitable access to acute care. Disparities in healthcare access between rural and urban areas highlight the need for targeted interventions. In a separate study by Gotsman et al., focusing on ischemic heart disease patients within a health maintenance organization, it was observed that the prevalence of ischemic heart disease was higher among younger individuals, particularly those residing in urban areas with obesity and diabetes^21^.

Despite similar mortality rates across all groups, differences were noted in cardiovascular hospitalizations and deaths. Thus, in order to mitigate these disparities, preventive initiatives targeting risk factors such as diabetes and obesity should be prioritized.

## Limitations

Due to the impact of COVID-19, the pace of data collection significantly slowed, resulting in a lag in updating information within the CDC WONDER database for the years 2021–2023. As a consequence, our analysis was constrained to documenting the most recent trends in mortality. Future research endeavors could explore these trends further once updated data becomes available.

In our analysis, age, gender, and race were the primary factors examined in relation to ischemic heart disease mortality, without delving into subcategories. However, it is important to note that social status and access to healthcare also play crucial roles in influencing mortality rates. Regrettably, these factors were not explored in our study due to limitations inherent in the CDC WONDER database, which does not encompass all contributing causes of mortality. Thus, they were not within the scope of our investigation.

Additionally, our focus was on comparing mortality proportions rather than modeling temporal trends or complex multivariate relationships. While our current approach provided valuable insights into disparities and trends, we acknowledge that applying time-series analyses, repeated measures regression, or multivariate modeling could yield deeper understanding of temporal patterns or causal inferences. Future studies could build upon our findings using these advanced methodologies.

The urbanization classification from 2013 was applied to the entire study period (1999–2020). This assumption could introduce bias, as some areas may have undergone classification changes over this period. Future research should consider integrating dynamic urbanization data to address this potential limitation.

Finally, the dataset used was directly extracted from the CDC WONDER database, which provides crude mortality rates but does not compute age-standardized mortality rates (ASMRs). Incorporating ASMRs would require additional population age-structure data and recalculations, which were beyond the scope of this study. However, we recognize the importance of age-standardization in minimizing confounding effects and plan to address this adjustment in future analyses.

## Conclusions

The study highlights significant disparities in mortality rates between urban and rural populations, with urban areas showing higher overall mortality rates. Within these populations, the elderly, particularly those aged 75 and above, are disproportionately affected, accounting for the highest mortality rates. This trend reflects the increased vulnerability of older individuals to chronic diseases and age-related health conditions. Gender differences are also evident, with males contributing more significantly to mortality rates, possibly due to a combination of biological factors and higher engagement in risk-prone behaviors. Racial disparities further complicate the picture, as white and African American populations exhibit elevated mortality rates compared to other racial groups. These findings underscore the multifaceted nature of mortality disparities, shaped by age, gender, geographic location, and race.

Various factors contribute to these mortality disparities, particularly in the context of ischemic heart disease. White and African American populations have notably higher mortality rates from this condition compared to Asian Americans, likely due to differences in lifestyle, healthcare access, and genetic predispositions. Higher rates of smoking, obesity, unhealthy diets, and physical inactivity in white and African American populations significantly increase their risk of ischemic heart disease. Meanwhile, Asian Americans may benefit from healthier dietary habits, lower smoking rates, and genetic factors that provide some level of protection against heart disease. Socioeconomic factors and healthcare accessibility also play crucial roles, as urban residents may face challenges like stress, pollution, and lifestyle changes that elevate heart disease risks. Addressing these disparities requires targeted interventions that consider the complex interplay of social, biological, and environmental factors. By focusing on these key areas, policymakers can work towards reducing mortality differentials and promoting equitable healthcare outcomes for all populations. Ultimately, this study contributes to a better understanding of the factors behind the disparities in mortality rates between urban and rural populations and highlights the urgent need for improvements and effective interventions to reduce mortality rates.

## Statements and declarations

**Ethical approval:** All data was sourced from the CDC-WONDER database, which is a publicly accessible database containing anonymized information, and hence, the present study was deemed exempt from ethical approval.

**Funding:** The authors received no funding for the present study.

## Competing interests

The authors declare no competing interests relevant to the content of this article.

## Author contributions

All authors contributed to the study conception and design. Material preparation, data collection and analysis were performed by Nithin Karnan, Abdirahman Hassan Abdirahman, Gaythri Kilaru, Asis Reet Kaur, Kaustav Majumder, and Layla Al Safadi Abou Al Fadel. The first draft of the manuscript was written by Nithin Karnan, Abdirahman Hassan Abdirahman, Gaythri Kilaru, Asis Reet Kaur, Kaustav Majumder, and Layla Al Safadi Abou Al Fadel; and all authors commented on previous versions of the manuscript. All authors read and approved the final manuscript.

## Acknowledgements

The authors acknowledge The Good Research Project in the successful completion of the manuscript.
